# Use of a district health information system 2 routine immunization dashboard for immunization program monitoring and decision making, Kano State, Nigeria

**DOI:** 10.11604/pamj.supp.2021.40.1.17313

**Published:** 2021-11-12

**Authors:** Dieula Delissaint Tchoualeu, Hashim Elzein Elmousaad, Lynda Uju Osadebe, Oluwasegun Joel Adegoke, Chimeremma Nnadi, Suleiman Ahmed Haladu, Sara Michele Jacenko, Lora Baker Davis, Peter Brian Bloland, Hardeep Singh Sandhu

**Affiliations:** 1 Global Immunization Division, Center for Global Health, United States Centers for Disease Control and Prevention, Atlanta, United States of America,; 2 National Emergency Operation Center (NEOC), AVRAM/US CDC, Pakistan,; 3 National Stop Transmission of Polio, African Field Epidemiology Network, Nigeria

**Keywords:** Routine immunization, data quality, data use, District Health Information System 2 (DHIS-2), routine immunization module, dashboard, National Stop Transmission of Polio, Nigeria

## Abstract

**Introduction:**

a district health information system 2 tool with a customized routine immunization (RI) module and indicator dashboard was introduced in Kano State, Nigeria, in November 2014 to improve data management and analysis of RI services. We assessed the use of the module for program monitoring and decision-making, as well as the enabling factors and barriers to data collection and use.

**Methods:**

a mixed-methods approach was used to assess user experience with the RI data module and dashboard, including 1) a semi-structured survey questionnaire administered at 60 health facilities administering vaccinations and 2) focus group discussions and 16 in-depth interviews conducted with immunization program staff members at the local government area (LGA) and state levels.

**Results:**

in health facilities, a RI monitoring chart was used to review progress toward meeting vaccination coverage targets. At the LGA, staff members used RI dashboard data to prioritize health facilities for additional support. At the State level, immunization program staff members use RI data to make policy decisions. They viewed the provision of real-time data through the RI dashboard as a “game changer”. Use of immunization data is facilitated through review meetings and supportive supervision visits. Barriers to data use among LGA staff members included inadequate understanding of the data collection tools and computer illiteracy.

**Conclusion:**

the routine immunization data dashboard facilitated access to and use of data for decision-making at the LGA, State and national levels, however, use at the health facility level remains limited. Ongoing data review meetings and training on computer skills and data collection tools are recommended.

## Introduction

Data use is defined as “the analysis, synthesis, interpretation and review of data for data-informed decision-making processes, regardless of the source of data” [1]. Improving the collection and use of routine immunization (RI) data such as vaccine logistics, vaccination coverage and sessions is needed to improve the overall performance of immunization programs, inform policy decisions and plan effective activities such as vaccination service delivery in resource-limited settings. Availability and use of high quality RI data are vital to increasing vaccination coverage, which in turn prevents vaccine-preventable disease outbreaks in resource-limited countries [2]. “Data quality” is typically interpreted across three dimensions: data collection process, characteristics of the collected data and data use [3]. However, data quality and data use are interrelated, because the relationship between these two elements is a self-reinforcing feedback loop, greater data use leads to improved data quality [1, 4]. Despite a growing desire by RI programs to encourage a culture of data use in the public health community, few studies have adequately addressed data quality and use [5].

### Evolution of Nigeria´s RI and data flow in Kano State, Nigeria

The National Health Management Information System (NHMIS) collects national health data across programs, including immunization. Before 2014, RI data were collected through the World Health Organization´s District Vaccine Data Management Tool (DVDMT), a Microsoft Excel-based data management system. Access to those data was restricted to higher officials, which limited their use by health professionals at the national, State and local government area (LGA, also known as district) levels. Additionally, the National Expanded Program on Immunization (EPI) faced many challenges caused by data submission delays and poor data quality. With the support of many partner organizations, Nigeria´s government adopted the District Health Information System (DHIS) 1.4 in 2006 and replaced it with DHIS-2 in 2010 to strengthen the NHMIS. Although DHIS-2 was adopted as the only platform for reporting routine health data nationwide in 2013, the government of Nigeria continue to use the NHMIS, a parallel system which did not capture all data required to monitor key indicators as outlined in the Accountability Framework for RI in Nigeria (AFRIN) for monitoring EPI performance in the National Routine Immunization Strategic Plan 2013-2015 [6]. This resulted in the parallel use of both DVDMT and DHIS-2 by health workers for reporting vaccination data.

**Figure 1:**
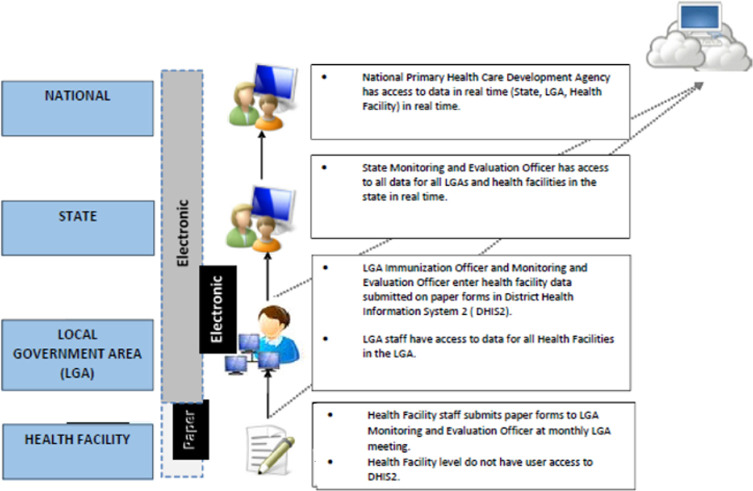
District heath information system 2 routine immunization data flow schema in Nigeria

**Figure 2:**
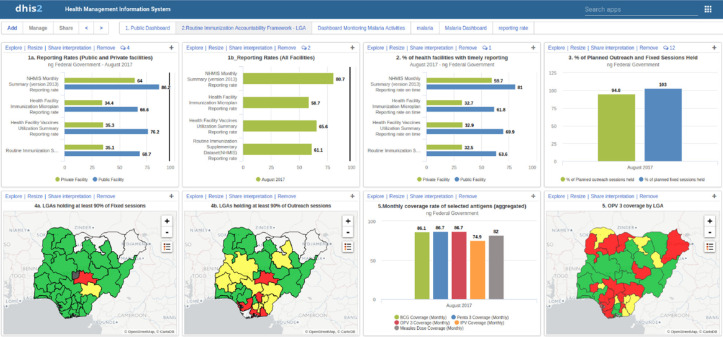
Screenshot of routine immunization dashboard output

In response, the National Primary Health Care Development Agency (NPHCDA), in collaboration with other immunization partners, piloted an RI module and dashboard to provide a unified platform for collection and display of all data required for monitoring RI performance indicators. This pilot was conducted in Kano State in November 2014. The RI module contains data required for measuring RI indicators that are not included in the NHMIS, as outlined in AFRIN such as vaccine usage, sessions planning and functionality of cold chain equipment. Consequently, the flow of RI data has changed significantly, as shown in Figure 1. Health Facility (HF) staff members use three paper forms to record RI data (NHMIS supplementary form, HF vaccine utilization summary form, HF microplan form), which they send to the LGA´s Monitoring and Evaluation (M & E) Officer for entry in the RI data module. At least 1 M & E officer was assigned to each LGA in Kano State to monitor LGA and HF level performance and track progress in reaching monthly and annual immunization target goals. The RI dashboard is an automated, real-time tool for visualizing and interpreting RI data for program monitoring and decision-making. Analyses of key RI indicators are automated, reducing the need to use analytical skills for visualizing the data. Figure 2 is a screenshot of the RI dashboard output. The State government mandated that all LGA officers use the dashboard module to review their respective immunization data monthly to monitor RI performance indicators at both the LGA and health facilities level. In 2018, the government made a full transition from the old information system (DVDMT) to DHIS2 RI module.

We assessed use of the RI module and RI dashboard to understand whether health care workers use RI data to inform programmatic decision-making at different levels of the national immunization program. Additionally, we sought to identify enabling factors and barriers to effective and timely use of RI data for program decision-making.

## Methods

A mixed method of quantitative and qualitative approach used for this assessment in 2016. The quantitative component of this study was integrated in an assessment of inactivated polio vaccine (IPV) introduction in Kano State, Nigeria [7] because multiple activities focused on the same target population (who were RI staff). It consisted of a survey questionnaire administered at the district and health facility levels. The qualitative method complemented the quantitative component of the study to gain in-depth understanding of the extent of RI data use in Kano State and how it is being used for decision-making. Desk reviews of program documents from all LGAs were also conducted to document data use and subsequent actions taken as a result of data use.

### Quantitative method: sampling, data collection, management and analysis

In brief, Kano State has 44 LGAs, 10 LGAs were randomly selected from 29 LGAs that introduced IPV before or during April 2015 (early IPV implementers) and 10 LGAs were randomly selected from 15 LGAs that introduced IPV after April 2015 (late IPV implementers). For the purpose of this study, findings were pooled for both groups and a total of 60 HFs (3 HFs/LGAs) were assessed [7]. Trainees in Nigeria´s Field Epidemiology and Laboratory Training Program administered a semi-structured interview questionnaire to 60 HF staff (RI officer or HF officer in charge) using either Open Data Kit (ODK) for mobile data collection or a paper-based form when use of ODK was not feasible. Four domains of questions were included in the questionnaire: 1) practices used for data quality monitoring and data review meetings; 2) practices used for supportive supervision visits; 3) use of RI data for program decision-making and 4) type of technical assistance needed to improve use of RI data for decision-making. National- and State-level officers of the National Stop Transmission of Polio Program (NSTOP) participated as supervisors or observers during data collection. Prior to data collection, data collectors and supervisors were trained on the purpose of the study, data collection tools and how to conduct semi-structured interviews. During the training, data collectors practiced among each other while a master trainer observed. Data from the semi-structured interview questionnaire were cleaned, validated and analyzed using Statistical Package for the Social Sciences (SPSS). The data were kept in a private cabinet file, only the investigators have accessed to the collected data. Descriptive analysis was conducted for all the variables (such as site demographics, number of supportive supervision visits, data review meetings and updating monitoring chart).

### Qualitative method: sampling, data collection, management and analysis

A convenience sample of 35 LGA immunization program staff members was selected for focus group discussions (FGDs) and in-depth interviews (IDI) from 10 sample LGAs. This convenience sample excluded personnel from the 20 LGAs used in the quantitative component of the study. FGD and IDI were conducted with partner organizations and key RI staff members at all levels to help the researchers understand strategies and best practices related to use of RI data for decision-making and perceived challenges encountered with RI data collection and use. Participants discussed their respective roles as LGA immunization officers (LIOs), monitoring and evaluation (M & E) officers, or NSTOP LGA Officers (NSLOs); general knowledge and use of the DHIS-2 RI dashboard; data use, enabling factors, and barriers related to general data use and those associated with using the RI dashboard and suggestions for improving the RI module and dashboard.

To prepare for the IDIs and FGDs, a two-day training on qualitative method was organized. Data collectors and supervisors learned how to conduct effectively IDI and FGD (including how to probe for additional information), how to prepare for qualitative data collection, how to moderate and manage FGD. Participants observed some mock interviews and practiced conducting interviews and FGD. This training also included basic elements of qualitative data analysis. A professional transcription service was used to transcribe field notes and recordings of IDIs and FGDs. Transcripts were reviewed initially by the principal investigator and then by two additional team members. The analysis was first conducted manually, coding and themes were organized in an Excel file. Different qualitative analytical techniques were used for the IDI and FGD data by “letting the data be the guide” of the analytic process [8]. Thematic analysis was conducted in an iterative manner based on a priori coding [9]. This analysis was facilitated by the *Sort & Sift, Think and Sift qualitative data analysis* approach [8]. It includes a number of strategies, such as extracting meaningful quotes from the IDIs and FGDs, “memoing” (i.e., summarizing investigators´ reflections of the data in the context in which it is examined), classifying themes and linking collected information from different sources of data to examine their relationships [8].

Desk reviews of program documents from all LGAs were conducted to document data use and action for program improvement. All available quarterly technical reports, monthly data review meeting minutes and reports on action taken to address issues identified during the data review meetings were reviewed. During the desk review, the principal investigator searched for key words and statements that discussed data quality and use, decision-making based on the RI data, issues identified to poor data quality and use, and action taken to address these issues.

### Ethical review

The United States Centers for Disease Control and Prevention (CDC) and the Kano State Medical Review Board determined this study to be public health program evaluation activity rather than human subject´s research. Participation in this assessment was voluntary. Participants provided verbal informed consent prior to data collection.

## Results

### Quantitative results

Of 60 HFs, 23 (38%) were rural health centers, 15 (25%) were government hospitals, 14 (23%) were health posts and 8 (13%) were other facilities. Sixty-three staff members were interviewed; 57% were heads of clinic and 43% were RI service providers.

#### Data use and supportive supervision at HF and LGA levels

All respondents from the HFs (N = 60) reported attending the monthly joint LIO/M & E meeting and most (N = 57) prepared for this meeting prior to attending (Table 1). The LIOs and NSLOs usually prepared for the LGA RI monthly review meeting by printing the dashboard page for each HF and distributing them to the respective HF members in attendance. During monthly review meetings, the HF personnel reviewed all of the dashboard pages to assess their performance and identify areas for improvement. This exercise helped introduce HFs to the RI dashboard. Although HF staff were responsible for providing the data that feed into the DHIS-2 system, they were generally not familiar with the DHIS-2 system and RI dashboard. In HFs, 62% (37/60) of those interviewed had heard about the RI dashboard and 47% (28/60) had seen it primarily through the monthly joint LIO/M & E meeting.

**Table 1:** advance preparation of health facilities for joint local government area immunization officers /monitoring & evaluation officers meeting in Kano State, 2016

Activities	Number (%)
Review routine immunization (RI) data only	33 (58)
Review RI data only and compile complaints/suggestions	9 (16)
Compile complaints/suggestions	6 (11)
Review RI data and follow up on any action plans from the previous meeting	1 (2)
Review RI data only, compile suggestions from previous meetings and follow-up on action plans	1 (2)
Other activities	7 (12)

Other activities include analyzing Pentavalent vaccine, identifying adverse events following immunization issues, ensuring that all materials are available, preparing tools, bringing with them RI data tools, and issuing monthly report.

RI staff members primarily used the manually developed RI monitoring chart as a visual tool to review progress toward meeting their target for vaccination coverage. Of 59 respondents, 56 (95%) knew how to use an RI monitoring chart. The staff member responsible for creating the chart was the RI provider (53%, n = 31), the HF in-charge (32%, n = 19), or another staff member (15%, n = 9). Most (51/59, 86%) of the HFs updated their RI monitoring chart on a monthly basis.

According to the reviewed RI data, all participating HFs received frequent supportive supervision visits, mostly conducted by representatives from the LGA (e.g., LIOs) and collaborating partners (Table 2). Documentation with written feedback was provided to HF staff in 93% (56/60) of supportive supervision visit sessions. During these visits, RI data from more than one of the reporting forms (such as from the micro-plan and vaccine utilization summary forms) and other relevant documents (e.g., defaulter´s list and past supervisory notes) were reviewed to identify data quality issues (e.g., discrepancies between tally sheets and registers and data entry errors) and to compare the number of planned immunization sessions to the number actually conducted.

**Table 2:** supportive supervision visits at the health facility level in Kano State, Nigeria, from March 2015-January 2016

Variable	Response	Frequency (%)
Number of supportive supervision visits		
	< 10 times	5 (8)
	10-20 times	15 (25)
	>20 times	40 (67)
Written report of visit	No	4 (6)
	Yes	56 (93)
Visit included RI data review	No	6 (10)
	Yes	54 (90)
Type of RI data reviewed	None	6 (10)
	At least one of the reports*	7 (12)
	More than one	42 (70)
	Other reportsβ	5 (8)
Health facility receiving feedback about its performance based on RI Summary data sent to LGA	No	6 (10)
	Yes	54 (90)
Frequency of receiving supportive supervision visits	Every Month	51 (94)
	Every Quarter	2 (4)
	No Response	1 (2)
Where was feedback received from	At the LGA review meeting	49 (91)
	During supportive supervision	5 (9)
Last time to receive feedback on RI summary data	Within the last month	48 (89)
	≥ 1 month ago	6 (11)

* Reports include: Micro plan, vaccine utilization summary, monthly HF vaccine utilization reporting form, defaulters list, past supervisory notes; β Others include reports from Clinton Health Access Initiative, Cold chain officer, Person from drug revolving fund, UNICEF/Seasonal Malaria Chemoprevention Results from the semi-structured interview questionnaire

#### Technical support needed at both HF and LGA levels

Although the health workers used RI data frequently to monitor EPI performance within their respective LGAs, the respondents believed that they were not prepared to fully interpret the information. Among 60 respondents at the HF level, 28 (47%) asked for training on data collection and recording, 27 (45%) preferred training on interpretation, 21 (35%) requested training on presenting data from the RI monitoring chart and 19 (32%) also requested training on data use. At the LGA level, all participants requested training on data generation, visualization, interpretation and use of geographic information systems and mapping.

### Qualitative results

The desk review showed that the Kano State government and partner organizations were strongly engaged during the planning and implementation stage of DHIS-2 in Kano. Prior to implementation, the Kano State government created a steering committee consisting of NPHCDA and partner organizations to provide leadership and technical oversights to this project. All Kano LGAs received training on the revised RI tools, RI module and dashboard. RI data use is facilitated through monthly data review meetings and supportive supervision. Thirty-five staff members participated in IDIs and FGDs. IDI participants included 8 RI focal persons, 5 LIOs and 3 members of the state RI Working Group (RIWG). Nine M & E officers and 10 NSLOs participated in the FGD. The main findings of this study were classified into four areas: 1) experience with RI data through the lens of its users; 2) enabling factors to data use; 3) barriers to data use; 4) knowledge and perception of the RI dashboard.

#### Experience with RI data through the lens of their users

RI data are used at various levels of the Kano health system and illustrative examples of the users´ experience at various levels are provided below.

##### HF level

HF staff members were responsible for providing quality RI data to feed into the DHIS-2 system. They used the monitoring chart for RI program decision-making. Actions taken for high dropout rate or low Penta3 coverage included compiling a list of defaulters, convening volunteer community mobilizers to help with defaulter tracking, providing health education messages on the importance of completing vaccination and determining reasons for mothers´ non-adherence to the immunization schedule.

##### LGA Level

Prior to RI module and dashboard implementation, LGA staff members (except the M & E officers) did not have easy access to immunization-related data. Consequently, they were not able to assess their performance. Now, LIOs, M & E officers and the NSLOs use the RI dashboard to track performance of HFs and to identify under-performing facilities based on key vaccine coverage and process indicators (e.g., dropout rate, planned vs. conducted sessions). They also use the data to decide which type of training is needed, determine the number of unimmunized children, assess functioning of the cold chain, identify stock-outs and advocate for support for RI services. During FGD, one of the NSLOs stated:

*“Before DHIS 2, data were analyzed manually [in Excel] and few indicators were chosen. [Using DHIS2] you can look at what percentage was covered from month to month throughout the year and, at every particular point in time, you can track a facility and know what it is doing. And this [makes it] easier because the data has been entered from the LGA, so anybody from the LGA State, zone and national can track it and see what is going on. You can look at dropout rates, [number of] supervision [visits] conducted, [and] view analysis for your LGA and their [health] facilities”*.

The LGA monthly technical forums presented an opportunity for the LGA staff (LIOs and M & E) to review and discuss RI data with HF staff members, resolve discrepancies in reporting rates between data sources, discuss data quality issues and harmonize the RI data with the state immunization officer. Collectively, participants from both IDIs and FGDs repeatedly stated that:

*“We want to use the data for action.... DHIS-2 facilitates data use for action”*.

##### State and partnership level

Prior to implementation of the DHIS-2 system, partner organizations obtained RI data from the World Health Organization (WHO), the only partner with direct access to DVDMT. Since the introduction of the RI module, RI data have become readily available, enabling informed decision-making based on real-time data. Partners viewed the RI dashboard as a “game changer” saying that it reinforced the accessibility and visibility of RI data and fostered accountability for data within the program. Interviewees at the State level as well as partners appreciated that “we can actually use the data and act upon it,” and that they can take ownership of it. They believe that DHIS-2 has fostered collaboration at the LGA level to address data quality and other RI issues (e.g. high dropout rate, limited or no outreach sessions) as a team. Consequently, Kano State developed a policy in 2015 requiring RI data and the dashboard to be reviewed on a monthly basis to identify critical areas for improvement at the LGA level. Major issues identified, such as low reporting rates, are reported to the Commissioner of Health (head of the State Ministry of Health) and the State Immunization Program Committee for necessary action. The partner organizations also used the DHIS-2 RI system to decide which HFs to target for monthly visits and to examine trends in the number of children immunized. One of the partners interviewed asserted the following:

*“...[DHIS-2] serves like a background check to look at particular HFs, where children are under-immunized, inquire what the problem is, and it gives us the opportunity to take action and make targeted monthly visits at the HFs. We only get that through DHIS-2, so it gives us an opportunity, for example, to look at why we have more children with Penta3 than Penta2”*.

RI data were also used during the RIWG meetings to inform and support programmatic decisions (such as conducting data validation, developing data quality improvement work plan, developing and disseminating DHIS2 RI bulletin) and to discuss any RI-data related issues across all levels of the health system. Appropriate interventions or actions were taken within a week to address data quality issues raised during the meeting, including identifying the person(s) responsible for implementing and following up on the actions. In conjunction with other implementing staff members (e.g., LIOs, NSLOs), the implementing partners also reported conducting joint supportive supervision visits to review performance of the HFs, take appropriate actions to correct issues identified and provide on-the-job training.

#### Enabling factors for RI data use

According to the respondents, the RI dashboard has become one of the major tools used for decision-making on the RI program within Kano State health system since its inception, although its use varies across the administrative levels. Key factors that helped establish routine use of RI data include the AFRIN, the accountability framework that outlines key indicators for monitoring immunization program performance and the ownership of the RI data-shifting ownership and management of health information system from partners to the Ministry of Health.

Four technical fora continue to facilitate use of the RI data, information sharing and building support of key implementing actors. The first is the LGA monthly review meetings between HF RI providers and LIOs to discuss RI data quality issues and overall performance of the HFs over the previous months. Second, monthly joint LIO/M & E meetings occur with senior state health management officials, LIOs, and M & E officers to review RI data and to identify problems regarding RI coverage and data collection, management and quality. Third, there are monthly state-level M & E working group meetings to discuss M & E issues reflected on the monthly RI dashboard. Last, bimonthly state RI working group meetings are held to discuss RI program performance, review the RI dashboard and make decisions based on LGA performance. Although these technical fora existed before the introduction of the RI module and dashboard, their usefulness was enhanced by greater access to data and data visualization, enabling participants to have a more focused agenda and develop actionable priorities. Training on use of the RI module and regular supportive supervision also helped facilitate the use of the RI dashboard. All respondents appreciated the quality of training received, they reported that it helped them to be computer literate and to know how to navigate the RI dashboard. Typical training includes, but is not limited to, RI data collection tools, pivot tables, data visualization, data capture and data quality. Regular supportive supervision visits for RI and quarterly data quality and data use reviews provided other venues for RI data to be examined and discussed at the HF level.

#### Barriers and challenges to use of RI data dashboard

Although the RI dashboard was generally thought to be user-friendly, participants reported some challenges with the system. Primary barriers included unreliable internet connectivity and low computer literacy among users, which affected their ability to use the system, especially at the LGA level. Reported challenges also included difficulty with correcting data entry errors once data had been entered in the system, reporting number of vials instead of number of doses on the vaccine utilization form and the lack of reliable current population denominator data. In addition, DHIS-2 data from the very few private health facilities are underreported. Because they are not funded by the government, they do not feel obligated to report their RI data.

## Discussion

This assessment sought to examine the use of RI data and the DHIS-2 RI dashboard for programmatic decision-making and action at all levels of the Kano State health system, as well as to identify potential enabling factors and barriers to the use of the system. The assessment suggests that the RI dashboard is frequently used at the State and LGA levels. The observed use of RI data is most likely due to improved access to the data and the data visualization made possible by the associated dashboard. Additionally, strategies put in place to facilitate the use of the RI data dashboard (such as training, technical forums and supportive supervision) were observed to have contributed to its acceptance. Availability of real-time data along with ongoing data review meetings seemed to have increased the demand for and use of RI data. This increase in demand is attributed to the commitment and involvement of all stakeholders to review data collection tools and the interaction with HF staff to solve problems with data reporting. Similar strategies were used in Zanzibar [10], Ghana and Sierra Leone during implementation of their DHIS-2 based systems [11].

Barriers and challenges to data use identified in this assessment align with findings documented in other countries such as Ghana [11] and Kenya [12]. Examples include users´ insufficient understanding of the data collection tools, the lack of reporting from private HFs and the use of population denominator data of unknown reliability. Furthermore, strengthening the technical capacity of staff members at various levels is an ongoing challenge. Staff members across all levels identified improving their overall technical capacity on data collection, analysis, quality, use, interpretation and presentation as a major need.

One of the major strengths of the study was its use of a mixed-methods approach. The qualitative inquiry complemented the quantitative component of the study, enabling the investigators to gain greater insight into strategies used to facilitate RI data use and to identify barriers to use of RI data and the RI dashboard. Another strength was the contribution of this study to building the capacity of staff members, those both directly and indirectly involved with DHIS-2, to assess their own program at various levels and gain a deeper knowledge of issues related to data quality and use.

Because of security challenges and the small number of LGA staff members available to interview, a convenience sample was used instead of the purposive sampling design originally planned for FGDs and IDIs. Additionally, a relatively small sample was used for the semi-structured questionnaire (N = 60); therefore, the results from Kano may not be generalizable to the rest of Kano State or elsewhere in Nigeria. It was not possible to collect data via phone. Local RI staff preferred to meet face-to-face for the interviews to maximize time and resources. Some components of the survey questionnaire required verification of certain information at the health facility level. For example, data collectors had to review the register for frequency of supportive supervision visits (including written feedback that the district staff provided to the HF staff) and data collection tools for completeness and accuracy.

Results of this assessment are based on self-reports from the LGA and HF staff and could not be independently verified. Furthermore, social desirability may play a factor in the results, given that NSTOP staff members supervised and observed data collection. Participants may have provided responses that they deemed “more socially acceptable” to portray a more favorable image of the reality in the field. Also, this study was conducted one year after implementation of the RI module and the long-term effect of the RI module and dashboard in the state was not assessed.

Data collection instruments were in English. Even though the data collection team consisted of local staff members who spoke both English and Hausa, questions occasionally required additional interpretation in some areas to facilitate understanding of what was being asked and some nuances may have been lost in translation. The impact of this issue on the results is unknown.

## Conclusion

Our study demonstrated that RI data are used for action and that corrective measures on data quality and use are taken quickly. The DHIS-2 platform has strengthened the supportive supervision structure by allowing staff members across the public health system to use current RI program data to guide the supportive supervision visits and as a mechanism for strengthening their technical capacity. Additional support on use of data collection and reporting tools and interpretation of data could be provided through ongoing supportive supervision visits, data review meetings, and training to further enhance and sustain the improvements seen with the introduction of the DHIS-2 module and dashboard.

### What is known about this topic

- The National Primary Health Care Development Agency (NPHCDA) in Nigeria, in collaboration with other immunization partners, developed a customized RI data module and dashboard and integrated it into DHIS2. The RI data module and dashboard piloted in Kano State in November 2014.

### What this study adds

- The RI data module and dashboard enabled access to and use of data for decision-making at the LGA (district), State and national levels in Kano State. RI data use is facilitated through monthly data review meetings and supportive supervision.
